# TGFβ2-induced outflow alterations in a bioengineered trabecular meshwork are offset by a rho-associated kinase inhibitor

**DOI:** 10.1038/srep38319

**Published:** 2016-12-07

**Authors:** Karen Y. Torrejon, Ellen L. Papke, Justin R. Halman, Magnus Bergkvist, John Danias, Susan T. Sharfstein, Yubing Xie

**Affiliations:** 1 Colleges of Nanoscale Science and Engineering, SUNY Polytechnic Institute, 257 fuller road, Albany, New York, 12203, USA; 2 Department of Ophthalmology, SUNY Downstate Medical Center, Brooklyn, New York, 11203, USA

## Abstract

Members of the transforming growth factor beta (TGFβ) cytokine family have long been associated with affecting several cellular functions, including cell proliferation, differentiation and extracellular matrix (ECM) turnover. Of particular interest to this work, TGFβ2 has been linked to most types of glaucomas as a potential fibrotic agent that can cause elevation of intraocular pressure (IOP). Given that the trabecular meshwork (TM) provides most of aqueous humor outflow resistance in the eye, an *in vitro* bioengineered human TM (HTM) model has been created and validated by analyzing effects of TGFβ2 on transcellular pressure changes and outflow facility. These changes were correlated with several biological alterations induced by this cytokine, including ECM production and overexpression of HTM-marker myocillin. Furthermore, this TM model has been used to extend current knowledge of gene expression of cytokines involved in TGFβ-induced ECM turnover over time. In particular, the ability for a ROCK-inhibitor to diminish the effect of TGFβ on TM was demonstrated. This work supports the notion that anti-fibrotic activities of ROCK-inhibitors could counteract the elevation of IOP and increased strain observed in glaucomatous TM.

The transforming growth factor β (TGFβ) family members play a key role in immune responses and tissue development such as inflammation, wound healing, extracellular matrix (ECM) accumulation, bone formation, cellular differentiation, and tumor progression^1^,^2^. The importance of TGFβ in ophthalmology is evident by its endogenous expression in the anterior segment, as well as its presence in the aqueous humor, which is responsible for chamber-associated immune deviation, a mechanism that protects the eye from inflammation and immune-related tissue damage. In particular, TGFβ appears to be involved in the pathogenesis of certain glaucomas^3^. For example, TGFβ2 is significantly elevated in the aqueous humor of patients with primary open-angle glaucoma (POAG)^4^ and induces pathological changes at the human trabecular meshwork (HTM) and optic nerve. In POAG, the sheath of connecting fibrils or plaques as well as ECM components that are present in the cribriform region of the HTM increase significantly, leading to elevated intraocular pressure (IOP)^5^. Treatment of cultured HTM cells with TGFβ2 could increase the expression of ECM proteins, fibronectin and tissue transglutaminase—an enzyme known to crosslink ECM proteins into complexes that can no longer be digested by proteinases^6^. In human eye organ-culture perfusion studies, TGFβ2 treatment reduced the outflow facility and increased ECM accumulation in the HTM, indicating an elevation in IOP^7^. Additionally, enzyme plasminogen activator inhibitor (PAI), which inhibits most matrix metalloproteinases (MMPs), was increased as a result of TGFβ2 treatments^8^. The fibrogenic effect caused by TGFβ2 is believed to be mediated, in part, by activated RhoGTPase/Rho-associated kinase (ROCK). ROCK-inhibitors are a novel potential class of glaucoma therapeutics offering distinct applications including reduced IOP, improved ocular blood flow, inhibition of postoperative scarring, and even neuroprotection^9^,^10^. With demonstrated effectiveness in animal models, several ROCK-inhibitors are currently undergoing Phase II and III clinical trials in glaucoma^10^,^11^. ROCK inhibitors can affect the contractile properties of HTM cells, α-smooth muscle actin (α-SMA) expression, ECM accumulation in the outflow pathway and aqueous humor outflow^9^,^10^,^11^,^12^. Several of these factors are also affected by TGFβ2, suggesting a common pathway. However, there is a knowledge gap on how TGFβ regulates the outflow facility of HTM mechanistically and how HTM-characteristic proteins are regulated in the presence of ROCK inhibitors.

We have previously reported a 3D bioengineered HTM model that recapitulates the ultrastructure, biological and physiological features of the *in vivo* HTM^13^. Here, to address the roles of TGFβ2, we first validated effects of TGFβ2 at clinically relevant concentrations using this model. Next, we investigated whether the effects of TGFβ2 are mediated by ROCK, analyzing actin expression pattern, ECM deposition, changes in HTM marker expression of myocilin and αB-crystallin, modulation of MMPs and cytokines induced by TGFβ2 in the absence or presence of a representative ROCK inhibitor (Y27632) in relationship to the regulation of outflow facility.

## Materials and Methods

### Primary Human Trabecular Meshwork Cell Culture

Primary TM cells were isolated from donor tissue rings discarded after penetrating keratoplasty and carried out in accordance with relevant guidelines and regulations. The SUNY Downstate IRB has reviewed the protocol and determined that work is IRB exempt. Isolation of the cells was under this IRB-exempt protocol approved by the SUNY Downstate IRB. Isolation and culture conditions were as previously described^13^,^14^. Before use in experiments, all HTM cell strains were characterized for expression of αB-crystalline and α-smooth muscle actin. HTM cells were initially plated in 75 cm^2^ cell culture flasks with 10% fetal bovine serum (FBS) (Atlas Biologicals, Fort Collins, CO) in Improved MEM (IMEM) (Corning Cellgro, Manassas, VA) with 1% 10 mg/mL gentamicin. Fresh medium was supplied every 48 h. Cells were maintained at 37 °C in a humidified atmosphere with 5% carbon dioxide until 80–90% confluence at which point cells were trypsinized using 0.25% trypsin/0.5 mM EDTA (Gibco, Grand Island, NY) and subcultured. At least three donors’ human primary cell cultures were used during experiments. All studies were conducted using cells before the 5^th^ passage.

### Scaffold Fabrication

SU-8 2010 photoresist (MicroChem Corp.) was used to develop free-standing porous microstructures that served as scaffolds on which primary HTM cells were cultured. Using photolithographic techniques and a chrome mask with defined micrometer-scale features, we fabricated SU-8 scaffolds as previously described^13^. Briefly, a release layer was spin-coated on the silicon wafer (substrate) and baked at temperatures between 120–150 °C. Photoresist was spin-coated on the substrate to a final thickness of <5 μm, then baked at 95 °C and cooled to room temperature. The photoresist was exposed to UV-light (140 mJ/cm^2^) through a chrome mask containing the desired pattern, baked at 95 °C and developed in PGMEA developer (MicroChem Corp.) SU-8 scaffolds with desired features were released from the substrate, washed with isopropyl alcohol, air dried and stored at room temperature. Scaffolds were mounted on aluminum rings, sterilized using 70% ethanol and coated with 1% gelatin before seeding HTM cells.

### 3D Culture of HTM Cells on Scaffolds and TGFβ2 +/− Y27632 treatment

To create 3D HTM constructs, 40,000 HTM cells were seeded on each microfabricated SU-8 scaffold, which was surrounded by an aluminum ring. After seeding, each scaffold was placed in a well of a 24-well plate and cultured in 10% FBS-IMEM for 14 days. The aluminum ring allowed suspension of the scaffold, preventing cell contact with the bottom of the well. Medium was changed every 2–3 days. By day 14, HTM cells-scaffold constructs formed a 3D structure^13^. Samples were serum-starved in 1% FBS for 48 hrs and then treated with TGFβ2 (2.5 ng/mL in 4 mM HCl, R&D systems, Minneapolis, MN), vehicle alone, ROCK inhibitor Y27632 (10 μM, Sigma Aldrich, ST. Louis, MO) or a combination of TGFβ2 (2.5 ng/mL) and Y27632 (10 μM) in 1% FBS-IMEM for 3, 6 and 9 days, as indicated.

### Perfusion Studies

A perfusion apparatus was used as previously described^13^. After 14 days in culture, 3D HTM constructs were serum-starved in 1% FBS and treated with TGFβ2, TGFβ2/Y27632, or vehicle alone for 9 days (as described above). Then 3D HTM samples were securely placed in the perfusion chamber and perfused at various rates for 6 hrs per flow rate (2, 10, 20 and 40 μl/min or 2, 4, 6, 8, 10 μl/min). Samples were perfused in an apical-to-basal direction with perfusion medium consisting of Dulbecco’s modified Eagle’s medium (DMEM) (Cellgro) with 0.1% w/v gentamicin (MP Biomedicals) containing vehicle, 2.5 ng/mL TGFβ2 or TGFβ2 with 10 μM Y27632. The temperature was maintained at 34 °C throughout the experiment. Pressure was continuously monitored and recorded. After perfusion, the outflow facility of our bioengineered 3D HTM model was calculated from the inverse of the slope of the pressure versus flow per unit surface area. At least eight different samples per condition, including HTM cells from three donors, were studied under perfusion. In later experiments, 10 μM Y27632 was also used as negative control.

### Phalloidin Staining, Immunocytochemistry, and Confocal Microscopy

3D HTM samples were fixed in 4% paraformaldehyde, permeabilized with 0.2% Triton X-100, and blocked using 5% goat serum. To reveal cytoskeleton arrangement, samples were stained for F-actin using phalloidin (ThermoFisher Scientific) and incubated with antibody against α-SMA (Sigma-Aldrich). To confirm HTM marker expression, samples were incubated with antibodies against HTM markers, myocilin and αB-crystallin, washed and incubated with their appropriate secondary antibodies. To examine ECM proteins, samples were incubated with antibodies against collagen IV, fibronectin, and laminin (Abcam), as described previously^13^. Samples were co-stained with DAPI to reveal cell nuclei, followed by confocal imaging. Laser scanning confocal microscopy was performed using a Leica SP5 confocal microscope, and images were acquired at 40X and 63X magnifications using an oil-immersion objective. Confocal images were processed using Leica LasAF software, and all confocal images within a given experiment were imaged and captured using the same laser intensity and gain settings in order to be able to compare intensities across samples.

### Protein Extraction and Western Blot Analysis

Cellular proteins were extracted with ice-cold radioimmunoprecipitation assay (RIPA) buffer (50 mM Tris-HCL, pH 7.5, 150 mM sodium chloride, 1% Triton X-100, 1% sodium deoxycholate, 0.1% sodium dodecyl sulfate, 25 mM NaF, 0.1 mM sodium orthovanadate, 10 mM NaP_4_O_7_, 1 nM phenylmethyl sulfonyl fluoride) containing protease inhibitors (Complete Protease Inhibitor, Roche, Manheim, Germany) on ice. Proteins were quantified by bicinchoninic acid assay (Thermo Fischer Scientific). 20 μg of proteins from each sample were separated by SDS polyacrylamide gel electrophoresis on a 4–12% gel in MOPS running buffer (ThermoFisher Scientific), transferred onto a PVDF membrane and probed with the following primary antibodies rabbit anti-myocilin (Sigma Aldrich), mouse anti-αB-crystallin, mouse anti-fibronectin, rabbit anti-collagen IV and mouse anti-β-actin (Abcam). HRP-conjugated goat anti-mouse or anti-rabbit secondary antibodies (Invitrogen) were used. Bound antibody was detected using FluorChem E (Protein Simple). Protein expression was analyzed by densitometry using ImageJ, and normalized to the housekeeping gene β-actin. All experiments were performed in duplicate for each of three donor cells.

### Quantitative Real-time PCR (qPCR) Analysis

Total RNA was extracted from samples cultured for 3, 5, and 9 days with TGFβ2, TGFβ2/Y27632, or without treatment using an RNeasy Plus Mini kit (Qiagen Inc., Valencia, CA). RNA concentrations were determined using a NanoDrop spectrophotometer. 20 ng of RNA per sample was used for each qPCR experiments. qPCR was carried out using TaqMan RNA-to-CT 1-Step Kit (Applied Biosystems, Carlsbad, CA) and performed on an AB StepOnePlus Real Time PCR system (Life Technologies) using primers for MMP2, MMP3, TIMP1, IL1α, TGFβ2 and GAPDH (Table 1). The temperature profile was as follows: 48 °C for 15 min (reverse transcription step), followed by an enzyme activation step of 95 °C for 10 min, 40 cycles of 15 s denaturation at 95 °C and 1 min anneal/extend at 60 °C. Relative quantitation data analysis was performed using the comparative quantification method, ΔΔCt, with GAPDH as the endogenous reference. All samples were normalized to the vehicle-treated controls. qPCR experiments were performed in triplicate (technical replicates) from duplicate biological experiments for each of the three donor cells. Average values are presented as mean ± SD.

### Statistical Analysis

Data are expressed as mean ± standard deviation. The difference between vehicle-treated (controls), TGFβ2-treated, and TGFβ2/Y27632-treated 3D HTM samples was analyzed using two-way ANOVA followed by Bonferroni post-tests (GraphPad Prism 6.02; GraphPad Software, Inc., La Jolla, CA). P values: P < 0.05, P < 0.01, and P < 0.001 are considered significant, very significant, and extremely significant, respectively.

## Results

### Validation of TGFβ2-induced physiological outflow changes in the 3D bioengineered HTM

**Figure 1:**
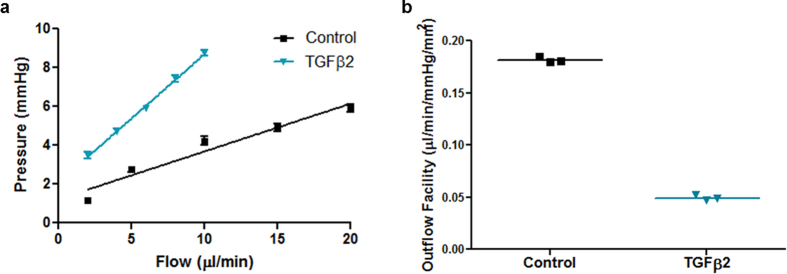
TGFβ2 increases outflow resistance in 3D HTM cultures after 9-day treatment. (**a**) Pressure change as a function of flow rate. (**b**) Calculated outflow facility for HTM cultures treated with vehicle control (square) or TGFβ2 (triangle).

Given that regulation of aqueous humor flow is one of the main functions performed by the HTM, we investigated the hydrodynamic behavior and transcellular pressure across the 3D HTM after treatment with TGFβ2 over at least 9 days. We utilized a perfusion system apparatus, as previously described^13^, that controls perfusion flow rate while constantly monitoring pressure changes. After treatment with 2.5 ng/mL TGFβ2 alone for 9 days, the bioengineered 3D HTM construct was place in the perfusion chamber and perfused with serum-free medium supplemented with 2.5 ng/mL TGFβ2 at various flow rates (2, 4, 6, 8 and 10 μL/minute) in an apical-to-basal orientation. Control samples were treated with vehicle and perfused at flow rates of 2, 5, 10, 15 and 20 μL/min in the apical-to-basal orientation. Higher flow rates were required for controls to consistently record pressure changes in the low range. For both treated and untreated samples, the pressure was continuously recorded for 6 hours to assure equilibrium at each flow rate. Compared to controls, TGFβ2 perfusion increased transcellular pressure (Fig. 1a) and significantly decreased (0.18 ± 0.03 versus 0.05 ± 0.01) the outflow facility per unit area across the 3D HTM (N = 10, P < 0.001) (Fig. 1b). These results show that our 3D HTM model is responsive to TGFβ-2 by drastically changing the transcellular pressure and outflow facility as expected.

### Validation of TGFβ2-induced F-actin rearrangements and co-localization of α-smooth muscle actin to stress fibers

**Figure 2:**
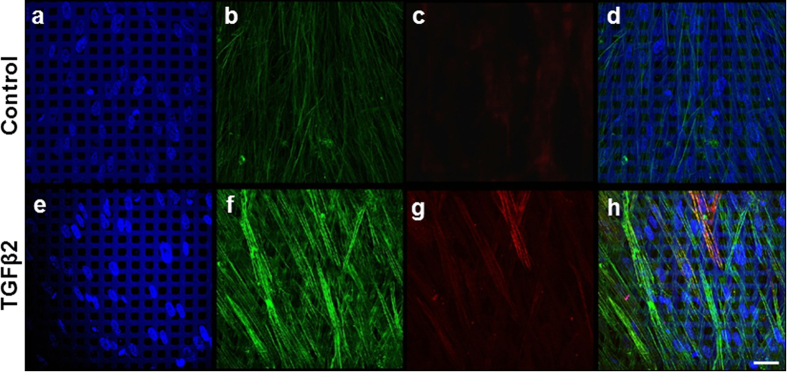
Confocal images of F-actin expression and α-SMA localization in 3D HTM cultures after TGFβ2 treatment for 9 days. (**a–d**) Vehicle-treated controls. (**e–h**) TGFβ2 treatment. (**a,e**) DAPI-stained nuclei in blue. (**b,f**) Phalloidin-stained F-actin in green. (**c,g**) Immunocytochemistry of α-SMA in red. (**d,h**) Merged images. Scale bar = 30 μm.

The cytoskeleton provides the framework for multiple cell functions including resisting deformation, coordination of forces that enable cell movement and control of shape^15^. Given the dynamic nature of the HTM, it is believed that HTM cells’ cytoskeletal alterations may allow the effective control of aqueous humor outflow resistance. We applied our previously established, 3D-bioengineered HTM model to study changes in actin arrangement upon TGFβ2 exposure. After primary HTM cells were cultured on gelatin-coated SU-8 scaffolds for 14 days, as described previously^13^, these 3D HTM constructs were conditioned overnight in 1% serum media and subsequently incubated for 9 days in the presence or absence of TGFβ2 (2.5 ng/mL) While cells in vehicle-treated controls exhibited aligned actin fibers, TGFβ2 treated samples showed increased staining of F-actin fibers that exhibited actin rearrangements and appeared more disorganized, despite seemingly aligned nuclei (Fig. 2). The actin expression was intensified in 3D HTM after treatment with TGFβ2. No significant change in nuclei size between vehicle-treated controls (35 ± 3 μm^2^, N = 20) and TGFβ-2 treated samples (38 ± 4 μm^2^, N = 15) was observed. Given previous reports of the induction of α-SMA by this cytokine *ex vivo* and *in vitro*^16^,^17^, we further studied the expression of α-SMA in our model. Our studies show an increase of α-SMA in 3D HTM after exposure to TGFβ2 and interestingly, a colocalization with F-actin fibers. While our controls expressed diffuse α-SMA in the HTM cytosol, after TGFβ2 treatment, this protein appears to be upregulated and arranged into fibers (Fig. 2g). These changes in actin arrangements could have a direct impact on cell behavior and cell interactions with the microenvironment, including the ECM and neighboring cells.

### Validation of TGFβ2-induced ECM deposition in bioengineered 3D HTM

**Figure 3:**
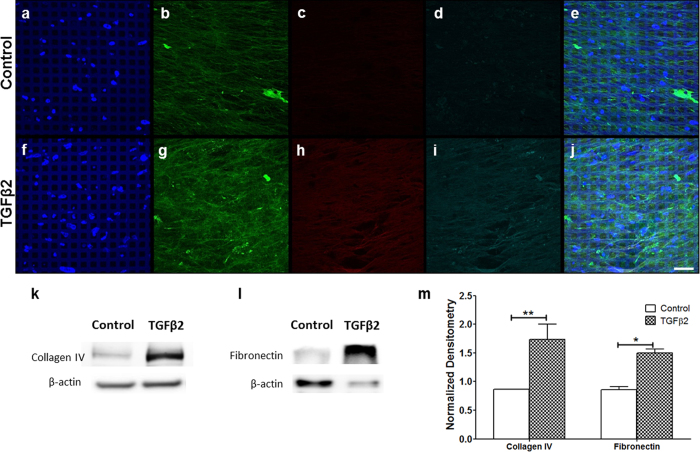
Induced expression of ECM proteins in 3D HTM cultures after perfusion with 2.5 ng/mL TGFβ2 for 9 days. (**a–j**) Confocal images of immunocytochemistry of 3D HTM perfused with vehicle control (**a–e**) or 2.5 ng/mL TGFβ2 (**F–J**). (**a,f**) DAPI-stained nuclei in blue. (**b,g**) Collagen IV in green. (**c,h**) Fibronectin in red. (**d,i**) Laminin in cyan. (**e,j**) merged images. (**k**) Representative western blots of collagen IV and β-actin cropped from the full-length blots displayed in Figure S1. (**l**) Representative western blots of fibronectin IV and β-actin cropped from the full-length blots displayed in Figure S2. (**m**) Densitometry of western blot analysis of collagen IV and fibronectin normalized to the housekeeping gene of β-actin. Scale bar = 30 μm. Asterisks indicate significance of difference from controls. **p < 0.01. *p < 0.05.

Increased interest in the pathogenic, fibrotic expression of several ECM proteins in glaucomatous HTM encouraged us to evaluate the effect of TGFβ2 on ECM protein expression in our bioengineered 3D HTM model. Fibrotic glaucomatous HTM is believed to result from the elevated amount of TGFβ2 found in the aqueous humor^3^,^18^. Exogenous treatment with TGFβ2 increased the expression of ECM proteins, collagen IV, fibronectin, and laminin, as shown by immunocytochemistry analysis (Fig. 3a–j). After treatment with TGFβ2, collagen IV fibers appeared highly disorganized and arranged into highly crosslinked bundles compared to vehicle-treated controls (compare Fig. 3b,g). Denser and thicker fibrous arrangements of fibronectin and laminin were seen after treatment with TGFβ2, in which multiple fibers joined together (fibrils) longitudinally, making fibers appear thicker and denser compared to those seen in controls (compare Fig. 3c,h, Fig. 3d,i). western blot analysis corroborated the induction of collagen IV (Fig. 3.k) and fibronectin (Fig. 3l) in 3D HTM cultures after TGFβ2 treatment and confirmed the significant increase in collagen IV (N = 4, P < 0.01) and fibronectin (N = 4, P < 0.05) proteins (Fig. 3m). All together, these results not only support the involvement of TGFβ2 in fibrotic HTM, as seen in several types of glaucomas, but also suggest the role of TGFβ2 in tissue hardening that may take place as a result of crosslinked, dense arrangements of collagen IV, fibronectin and laminin.

### Induced expression of HTM-marker protein myocilin but not αB-crystallin in 3D HTM after extended exposure to TGFβ2

**Figure 4:**
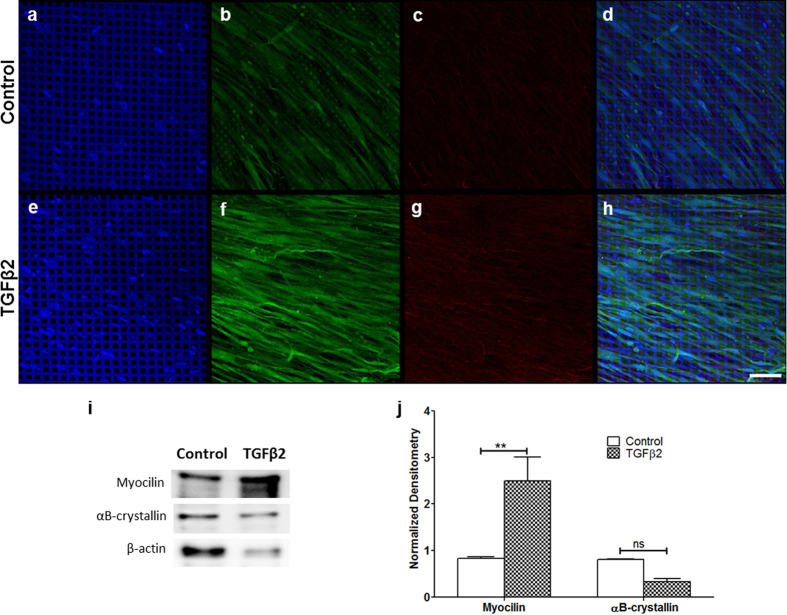
Expression of HTM marker proteins in 3D HTM cultures after treatment with 2.5 ng/mL TGFβ2 for 9 days. (**a–h**) Confocal images of immunocytochemistry of myocilin (green) and αB-crystallin (red) after treatment with vehicle control (**a–d**) or 2.5 ng/mL TGFβ2 (**e–h**). Scale bar = 100 μm. (**i**) Representative western blots of myocilin, αB-crystallin and β-actin cropped from the full-length blots displayed in Figure S2. (**j**) Densitometry analysis of western blot of myocilin and αB-crystallin, normalized to β-actin. Asterisks indicate significance of difference from controls. **p < 0.01. n.s.: p ≥ 0.05.

Despite decades of research in the field, myocilin and αB-crystallin are two characteristic, but poorly understood proteins that have been found in the HTM. Myocilin, a secreted protein known to interact with the ECM, apparently causes disease only in the eye^19^. αB-crystallin is a small heat shock protein with chaperone activity found mostly in the cribriform region of the HTM, the area adjacent to the inner wall of the human Schlemm’s canal (HSC), which is believed to exert the most pressure regulation^20^. αB-crystallin expression increases after a few days of exposure to TGFβ2^21^. However, the effects of extended exposure to TGFβ2, e.g., a week or more, are unclear. We studied the effects of prolonged (9 days or more) TGFβ2 exposure on the expression of myocilin and αB-crystallin using immunocytochemistry (Fig. 4a–h) and western blot analysis (Fig. 4I,j). Confocal imaging showed an increase in myocilin protein expression in 3D HTM cultures after TGFβ2 perfusion (Fig. 4f) compared to vehicle-treated controls (Fig. 4b).

The myocilin accumulation, induced by TGFβ2, appeared punctate and occasionally plaque-like in areas between cells (Fig. 4F). On the other hand, 3D HTM cultures treated with TGFβ2 showed no change in αB-crystallin expression (Fig. 4g) compared to control (Fig. 4c). These results were confirmed by western blot analysis (Fig. 4I) in which the densitometry analysis showed that expression of myocilin after TGFβ2 treatment was significantly higher than control (N = 4, P < 0.01) while there was no significant difference in expression of αB-crystallin (N = 4, P > 0.05) (Fig. 4j).

### Suppression of TGFβ2-induced overexpression of ECM proteins and myocilin in 3D HTM by a ROCK inhibitor

**Figure 5:**
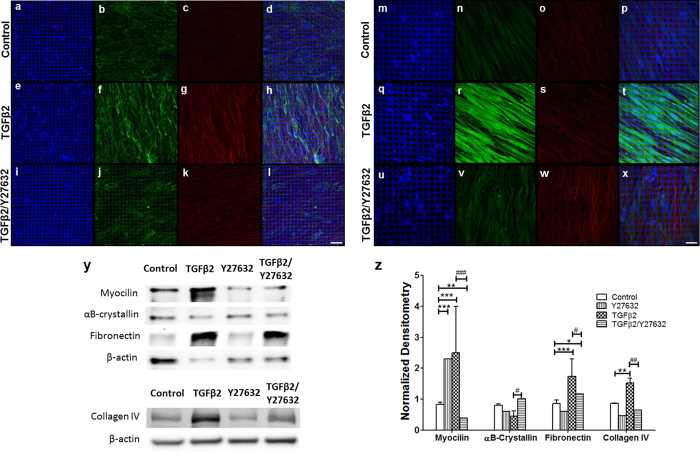
Confocal images of ECM and HTM-markers protein expression in 3D HTM cultures after treatment with 2.5 ng/mL TGFβ2 in the absence or presence of 10 μM Y27632 for 9 days. (**a–d**,**m–p**) Vehicle control. (**e–h**,**q–t**) TGFβ2. (**i–l,u–x**) TGFβ2/Y27632. (**a,e,i,m,q,u**) DAPI-stained nuclei in blue. (**b,f,j**) Collagen IV (green). (**c,g,l**) Fibronectin (red). (**d,h,l**) merged images. Scale bar = 50 μm. (**n,r,v**) myocilin (green). (**o,s,w**) αB-crystallin (red). (**p,t,x**) merged images. Scale bar = 30 μm. (**y**) Representative western blots of myocilin, αB-crystallin, fibronectin, and respective β-actin (top panel); and collagen IV and its respective β-actin (bottom panel) for 3D-HTM scaffolds treated with vehicle-control, 10 μM Y27632, 2.5 ng/mL TGFβ2, or 2.5 ng/mL PA/10 μM Y27632 for 9 days, which were cropped from the full-length blots displayed in Figure S1 and S2. (**z**) Densitometry analysis of western blot. Asterisks indicate significance of difference from controls ***p < 0.001, **p < 0.01, *p < 0.05. Hash marks indicate significance of difference from TGFβ2 treatment ^###^p < 0.001, ^##^p < 0.01, ^#^p < 0.05.

ROCK inhibitors are currently being considered as a potential new class of glaucoma drugs. Despite extensive studies on their effects on the HTM, it is not clear whether ROCK inhibitors can correct TGFβ2-induced fibrotic ECM build-up and myocilin over-expression, which have been linked to glaucoma. Therefore, we assessed the effect of co-administering TGFβ2 along with a ROCK inhibitor, Y27632, on ECM protein secretion and myocilin expression. Immunocytochemistry analysis of our bioengineered 3D HTM model co-treated with TGFβ2/Y27632 for 9 days showed substantially decreased collagen IV and fibronectin expression compared to samples treated with TGFβ2 alone (Fig. 5a–l). In the Y27632 co-treated samples, shorter collagen IV fibers and more aligned fibronectin fibers (Fig. 5i–l) were observed compared to samples treated with TGFβ2 alone (Fig. 5e–h).

Similarly, treatment with TGFβ2/Y27632 combined, significantly reduced the expression of myocilin while slightly increasing the expression of αB-crystallin (Fig. 5u–x) compared to treatment with TGFβ2 alone (Fig. 5q–t). western blot analysis further confirmed these immunocytochemistry results, showing that TGFβ2/Y27632 treatment caused a significant decrease in ECM proteins, fibronectin (N = 5, P < 0.05) and collagen IV (N = 5, P < 0.01 for all) in comparison to TGFβ2 alone, to the level of vehicle-treated controls or Y27632-treated samples (Fig. 5y), demonstrating that the presence of ROCK inhibitor could prevent the ECM accumulation caused by TGFβ2. Interestingly, treatment with either TGFβ2 or Y27632 alone induced overexpression of myocilin (Fig. 5z). This increase in myocilin expression was significantly suppressed by combined TGFβ2/Y27632 treatment when compared to vehicle-treated controls (N = 6, P < 0.001) and TGFβ2 samples (N = 6, P < 0.001) (Fig. 5z.). As mentioned previously, TGFβ2 treatment slightly, but not significantly, decreased αB-crystallin expression (Fig. 4j). The presence of Y27632 during TGFβ2 treatment significantly increased αB-crystallin expression (N = 6, P < 0.05), bringing it back to the level of vehicle-treated control or Y27632-treated samples. Compared to vehicle treated controls, TGFβ2/Y27632 combined treatments decreased myocilin (N = 6, P < 0.01) and increased fibronectin (N = 5, P < 0.05) expression (Fig. 5y,z). These results demonstrate that TGFβ2 combined with the ROCK inhibitor counteracts the otherwise fibrotic effect of TGFβ2, effectively lowering ECM protein accumulation and myocilin expression.

### ECM Remodeling through MMPs and Cytokines in 3D HTM treated with TGFβ2 in the absence or presence of ROCK inhibitor

**Figure 6:**
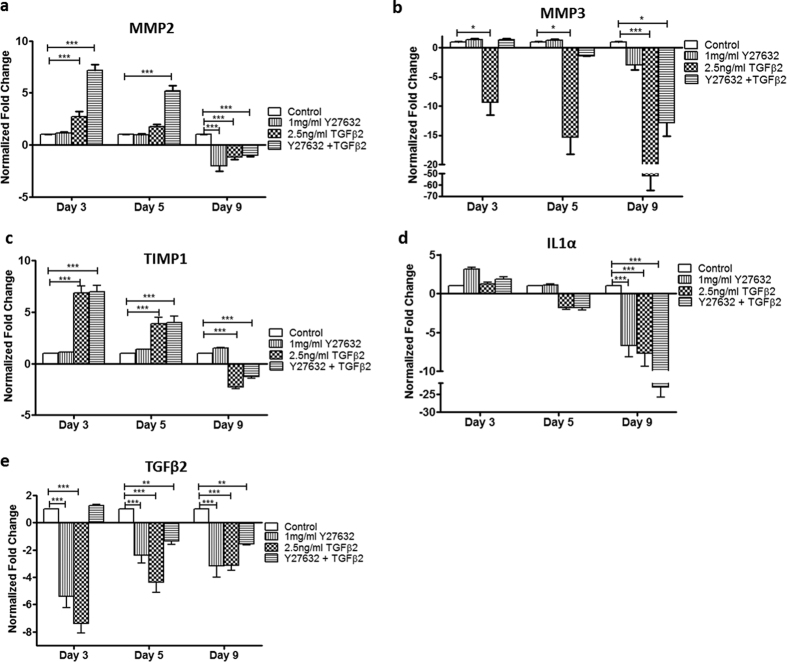
qPCR analysis of gene expression of MMPs and cytokines in 3D HTM cultures after 3, 5 and 9 days of treatments with TGFβ2 and/or ROCK inhibitor (Y27632). (**a**) MMP2. (**b**) MMP3. (**c**) TIMP1. (**d**) IL1α. (**e**) TGFβ2. Asterisks indicate significance of difference from controls ***p < 0.001, **p < 0.01, *p < 0.05.

To explore the factors that may be responsible for modulation of the TGFβ induction of ECM and other secreted proteins by ROCK inhibitor, the transcriptional expression of several MMPs and cytokines, including tissue inhibitor of metalloproteinase-1 (TIMP1), interleukin 1-alpha (IL1α) and TGFβ2, were evaluated through a 9-day time course study. MMP2 gene expression increased ~2.7-fold after a 3-day TGFβ2 treatment compared to vehicle-treated controls (P < 0.001) (Fig. 6a). This increase was further enhanced to ~7.1-fold in TGFβ2/Y27632 co-treatment while Y27632 treatment alone did not change MMP2 gene expression. After 5 days in culture, TGFβ2 treatment showed no significant difference in MMP2 gene expression from vehicle-treated control while in TGFβ2/Y27632 co-treated samples, MMP2 gene expression remained high (~5.1-fold increase compared to control, P < 0.001). Interestingly, MMP2 expression decreased after 9 days treatment with Y27632, TGFβ2, or TGFβ2/Y27632 (P < 0.001 for all treatments compared to control) (Fig. 6a). MMP3 expression showed no significant difference between control and TGFβ2/Y27632 treated cultures at day 3, while down-regulation in TGFβ2-treated samples (~9.4-fold decrease, P < 0.001) was observed. By day 5, MMP3 expression was down-regulated ~15.3-fold (P < 0.001) for TGFβ2 treatment compared to control; the TGFβ2 downregulation was suppressed by co-treatment with Y27632 (Fig. 6b). By day 9 of treatment, TGFβ2 and TGFβ2/Y27632 significantly decreased MMP3 mRNA expression by ~52- (P < 0.001) and ~13-fold (P < 0.05), respectively.

HTM cells secrete several cytokines that modulate cell behavior and ECM turnover in the conventional outflow pathway. Increased cytokine expression has been observed in the aqueous humor of glaucoma patients, including TIMP1^22^ and TGFβ^23^,^24^. In addition, IL1α is an inflammatory cytokine that has been shown to be involved in regulating outflow facility^25^. Therefore, studying cytokine expression could provide insight into the pathology of glaucoma as pertaining to TGFβ2. TIMP1 mRNA expression was up-regulated ~7- fold after 3 days of TGFβ2 (P < 0.001) or TGFβ2/Y27632 (P < 0.001) treatment. On day 5, TIMP1 gene expression levels were still higher than controls, ~4-fold increased by TGFβ2 (P < 0.001) or TGFβ2/Y27632 combined (P < 0.001). By day 9, TIMP1 was down-regulated by ~2.5-fold (P < 0.001) after TGFβ2 and by ~1.5 (P < 0.001) in TGFβ2/Y27632 treatments (Fig. 6c). Y27632 treatment alone did not affect expression of TIMP1, nor did the presence of Y27632 change TGFβ2-induced gene expression of TIMP1. On the other hand, IL1α gene expression exhibited no significant change after treatment with Y27632, TGFβ2, or Y27632/TGFβ2 for 3 and 5 days (Fig. 6d). On day 9, IL1α mRNA expression decreased by ~7.7-fold for TGFβ2 treated samples (P < 0.001) while co-treatment with Y27632 during TGFβ2 treatment further down-regulated this gene by ~23-fold (P < 0.001) (Fig. 6d). Both treatments exhibited a time-dependent effect on IL1α mRNA expression. As TGFβ2 can also be produced and secreted by HTM cells, we evaluated its expression in the presence of exogenous TGFβ2. TGFβ2 gene expression was down-regulated throughout days 3, 5 and 9 of exogenous TGFβ2 treatment by 7.4-, 4.4- and 3.2-fold, respectively. TGFβ2/Y27632 co-treatment initially (day-3) up-regulated TGFβ2 gene expression by 1.3-fold and later, during 5 and 9 days of treatment, down-regulated TGFβ2 mRNA expression by 1.4- and 1.6-fold, respectively, compared to control (Fig. 6e); however, in comparison with TGFβ2 treatment alone, the expression in the co-treated cultures was much closer to control levels. Overall, these data demonstrate a complex ECM regulation that takes place as a result of elevated exogenous TGFβ2 or TGFβ2/Y27632 exposure at the TM.

### ROCK inhibitor prevents TGFβ2-induced elevated flow resistance

**Figure 7:**
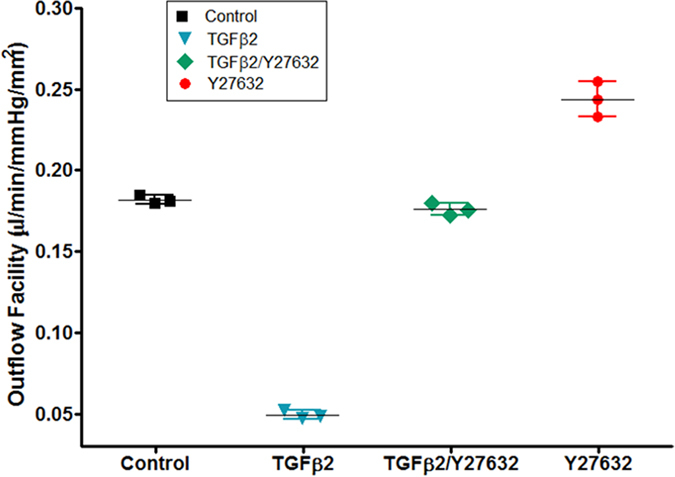
ROCK inhibitor neutralizes TGFβ2-induced decrease of outflow facility in 3D HTM. Calculated outflow facility of perfused vehicle control (square), TGFβ2 (inverted triangle), TGFβ2/Y27632 (rhombus), and Y27632 (circle).

Given the dramatic changes in protein and cytokine expression resulting from the combined treatment with TGFβ2 and ROCK inhibitor, we examined the effect of combined treatment on outflow facility through perfusion studies. As Y27632 alone increases outflow facility, we compared vehicle-treated controls, TGFβ2 treatment, Y27632 alone, and Y27632 in conjunction with TGFβ2 in 3D HTM cultures. Compared to vehicle-treated controls, TGFβ2/Y27632 perfusion maintained a similar outflow facility as shown in Fig. 7 (N = 6, ns). Vehicle- and Y27632-treated cultures showed an outflow facility of 0.184 ± 0.003 μL/min/mmHg/mm^2^ and 0.23 ± 0.04 μL/min/mmHg/mm^2^, respectively. On the other hand, TGFβ2 treatment significantly lowered the outflow facility of the bioengineered 3D HTM cultures to 0.049 ± 0.004 μL/min/mmHg/mm^2^ (P < 0.001) and TGFβ2/Y27632 treatment returned the outflow facility to 0.176 ± 0.008 μL/min/mmHg/mm^2^ (Fig. 7). These data demonstrate that the bioengineered 3D HTM regulates flow in a similar fashion as aqueous humor outflow regulation seen in *in vivo* and *ex vivo* HTM after TGFβ2 treatment, by increasing pressure and decreasing the outflow facility across this tissue. In addition, combined TGFβ2/Y27632 treatment maintained “normal” outflow regulation comparable to the vehicle-treated bioengineered HTM. Altogether, these data demonstrate that TGFβ2 is capable of modulating outflow of the bioengineered 3D HTM comparably to *in vivo* and *ex vivo* HTM, and the presence of ROCK inhibitor Y27632 during TGFβ2 treatment can prevent TGFβ-2-induced elevation of transcellular pressure and maintain the outflow facility close to control levels. These data provide insight into the use of ROCK inhibitors to treat elevated IOP in glaucoma patients who have elevated TGFβ2 in their aqueous humor.

## Discussion

In this study, we demonstrated that was our previously established bioengineered 3D HTM model^13^ responds to TGFβ2 treatment similarly to *in vivo* or *ex vivo* HTM, including elevated transcellular pressure, decreased outflow facility, and increased ECM accumulation. These data further validate the utility of the 3D HTM model for perfusion studies of biological agents believed to affect the TM, in a physiologically relevant setting, which are otherwise difficult to conduct using *ex vivo* organ cultures. Using a bioengineered 3D HTM model system, we further revealed that TGFβ2 affects a range of proteins that can elicit tissue stiffening and outflow facility changes, including ECM proteins, F-actin, and α-SMA. Lastly, we explored the potential of ROCK inhibitor in conjunction with TGFβ2 to rescue the homeostatic behavior of HTM through MMPs and inflammatory cytokines.

The actin cell machinery polymerizes to form different network organizations that provide cells with mechanical elements and properties^26^. Filamentous actin such as stress fibers also connects the cytoskeleton to the ECM via focal adhesion sites. Treatment with TGFβ2 caused cytoskeletal actin changes including polymerization of F-actin fibers and increased expression of α-SMA. After treatment, a majority of these F-actin fibers appeared to be organized into bundles, F-actin fibers in close proximity to other fibers. Actin bundles have innate compressive mechanical properties that can be measured by their buckling force, which is directly proportional to the number of actin filaments that make up a bundle^26^. In this way, tissues can increase their compressive force/strength through their actin arrangement. Based on our results, the induction of actin bundles suggests a more rigid cytoskeleton arrangement that can provide greater compressive forces to their microenvironment. Treatment of our 3D HTM model with TGFβ2 for 9 days also induced α-SMA overexpression and its rearrangement. α-SMA is a mechanosensitive protein, reported to be induced in response to critically stiffened cell mircroenvironments^27^ and by exogenous TGFβs. Furthermore, α-SMA is co-localized or recruited to stress actin fibers under high tension, and localization of this protein from the cytosol to the stress fibers can be mediated under specific topographic cues that affect the structure of focal adhesions^28^. In this study, we observed increased expression of ECM proteins collagen IV, fibronectin and laminin, along with elevated expression of α-SMA after TGFβ2 exposure. These induced ECM proteins together could substantially increase the stiffness of the immediate cell substrate, inducing the co-localization of α-SMA to the stress actin fibers as observed and previously discussed^29^,^30^. Furthermore, fibronectin, via feedback activation, induces α-SMA expression^16^. Therefore, the increased expression of fibronectin seen in this work could also be involved in the increased α-SMA expression. Collectively, these observations further support the interplay between HTM cell contractile hardware and ECM synthesis/deposition in the aqueous humor outflow pathway. In addition, the elicited expression of α-SMA and ECM proteins by TGFβ2 suggests transdifferentiation of HTM cells into myofibroblast-like cells expressing fibrogenic and fibroblast-like markers, as recently reported by others^31^,^32^.

Increased ECM deposition in the TM is associated with POAG. Cell-ECM interactions play a key role in regulating outflow in the eye^33^,^34^. The overproduction of ECM components such as fibronectin and collagen IV by HTM cells under pathological conditions has been described previously. For instance, we have shown that steroid treatment upregulates secreted and cell-associated ECM proteins, including fibronectin, collagen type IV, and laminin in a 3D bioengineered HTM model. Exposure to high glucose concentrations also induces fibronectin production by HTM cells^35^. In this work, ECM overproduction, including collagen IV, fibronectin, and laminin, was induced by TGFβ2. These proteins are the major components of the HTM basement membrane and are found throughout the conventional outflow tract. ECM proteins are dynamically remodeled by several enzymes including MMPs. HTM cells constitutively express significant levels of MMPs, such as stromelysin-1 (MMP3) and gelatinase A (MMP2); expression can be further elevated in response to a variety of stimuli such as mechanical stretching and biologically active cytokines^36^,^37^. Exposure to TGFβ2 enhances accumulation of the pro form of MMP-3 and decreases active MMP3 in the TM^38^. The unbalanced expression of MMP3 may result in decreased proteolytic degradation and increased accumulation of ECM components such as fibronectin, a known MMP3 substrate^39^. Indeed, in this study, exposure to TGFβ2 led to a significant increase in fibronectin expression and decreased gene expression of MMP3. In addition, MMP2 expression increased after 3 and 5 days of TGFβ2 exposure, which is consistent with previous short-term studies^40^. It is important to highlight that over an extended 9 days of treatment, MMP2 mRNA expression decreased, dropping well below the control levels. The initial increase in MMP2 was accompanied by TIMP1 induction. A similar relationship between MMP2 and TIMP1 has been shown after cyclic mechanical stretching of bovine TM cells^41^. In our model, TGFβ2 induces actin and ECM changes that can exert biomechanical stress on HTM cells leading to ECM turnover, accompanied by modulating MMP and cytokine expression, which is attenuated during longer term exposure to TGFβ2.

The initial (day 3) up-regulation of TIMP1, along with the sustained down-regulation of MMP3 during TGFβ2 treatment could account for the accumulation of ECM proteins. The early upregulation of MMP2 suggests an active ECM remodeling during early TGFβ2 exposure, perhaps due to TM cells attempting to counteract ECM protein overproduction. With extended exposure to TGFβ2, MMPs and TIMPs are down-regulated, potentially leaving considerable paracellular accumulation of ECM proteins and myocilin at the HTM basement membrane. A similar trend in MMP gene expression was observed after extended TGFβ2/Y27632 co-treatment, but towards the beginning (day 3–5) MMP2 mRNA-level was enhanced to a greater extent (over 2-fold) compared to TGFβ2 treatment alone. This potent induction of MMP2 gene expression could be sufficient to prevent or even disrupt proper assembly of proteins including myocilin and/or ECM proteins that require focal-adhesions. By the same token, given that IL1α has been shown to regulate MMPs in the TM or organ cultures^25^, down-regulation of this gene could induce the down-regulation of MMPs seen towards later days in this study.

**Figure 8:**
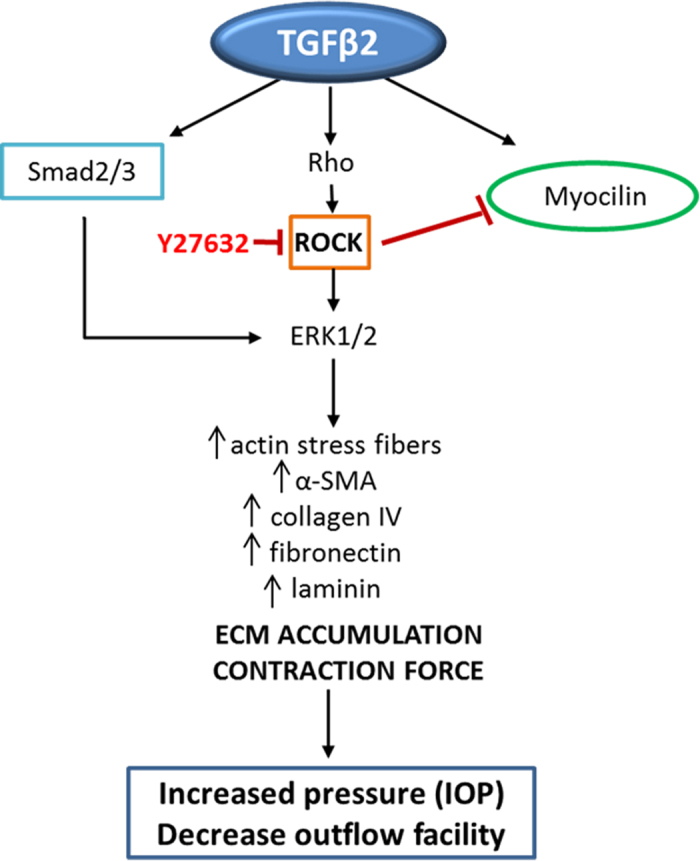
Schematic illustration of the effects elicited by TGFβ-2 that may induce IOP elevation. ROCK-signaling appears to regulate ECM synthesis/deposition and contractile activity by actin modulations. In the presence of TGFβ2, bidirectional molecular interplay, potentially transcriptionally independent, appears to takes place between ROCK and myocilin.

RhoA/Rho kinase signaling activity affects the expression of ECM proteins and αSMA^16^, in addition to its well-recognized role in actin cytoskeletal organization, and several ROCK inhibitors are currently undergoing Phase II and III clinical trials in glaucoma^9^,^10^. Using ROCK inhibitor, Y27632, in combination with TGFβ2, we assessed its effects on the expression of collagen IV, fibronectin, laminin, myocilin and αB-crystallin. This combination significantly decreased expression of ECM proteins as well as myocilin back to vehicle-treated controls levels, but failed to reduce αB-crystallin expression. Interestingly, treatment with Y27632 alone for 9 days upregulated myocilin and maintained unchanged levels of αB-crystallin compared to controls (Fig. 5). These results reinforce the anti-fibrotic nature, as related to ECM proteins, of ROCK inhibitors. On the other hand, these findings raise questions as to the relationship between myocilin, the ROCK pathway, and TGFβ2 and elucidate their probable interplay. Based on this work and current literature, there are several changes caused by TGFβ2 that may induce TM stiffening, eliciting elevated IOP (Fig. 8). Overall, this work supports the idea that misregulated accumulation of ECM proteins, and perhaps myocilin, may influence the biomechanical properties and stiffness of TM cells, and therefore, aqueous humor outflow facility.

## Additional Information

**How to cite this article**: Torrejon, K. Y. *et al*. TGFβ2-induced outflow alterations in a bioengineered trabecular meshwork are offset by a Rho-associated kinase inhibitor. *Sci. Rep.* **6**, 38319; doi: 10.1038/srep38319 (2016).

**Publisher's note:** Springer Nature remains neutral with regard to jurisdictional claims in published maps and institutional affiliations.

## Supplementary Material

Supplementary Information

## Figures and Tables

**Table 1:** qPCR primers.

Gene	Sequence
*MMP2*	5′-CCAAGGTCAATGTCAGGAGAG-3′
	5′-GCACCCATTTACACCTACAC-3'
*MMP3*	5′-TGAGTGAGTGATAGAGTGGT-3'
	5′-TGAACAATGGACAAAGGATACAAC-3'
*TIMP1*	5′-GCTTGGAACCCTTTATACATCTTG-3'
	5′-CCTTCTGCAATTCCGACCT-3'
*IL1A*	5′-AGTTCTTAGTGCCGTGAGTTTC-3'
	5′-GTGACTGCCCAAGATGAAGA-3'
*TGFB2*	5′-ACTTTGCTGTCGATGTAGCG-3'
	5′-GCAGAGTTCAGAGTCTTTCGT-3'
*GAPDH*	5′-TGTAGTTGAGGTCAATGAAGGG-3'
	5′-ACATCGCTCAGACACCATG-3'
