# Supplementary material for: TGFβ2-induced outflow alterations in a bioengineered trabecular meshwork are offset by a rho-associated kinase inhibitor
Source: Sci Rep. 2016 Dec 7;6:38319. doi: 10.1038/srep38319 (PMC5141429; doi:10.1038/srep38319)
Supplement: Supplementary Information [file srep38319-s1.doc]

**TGFβ2-induced outflow alterations in a bioengineered trabecular meshwork are offset by a rho-associated kinase inhibitor**

Karen Y. Torrejon^a^, Ellen L. Papke^a^, Justin R. Halman^a^, Magnus Bergkvist^a^, John Danias^b^, Susan T. Sharfstein^a^, Yubing Xie^a,*^

^a^Colleges of Nanoscale Science and Engineering, SUNY Polytechnic Institute, 257 Fuller Road, Albany, New York, 12203, USA

^b^Department of Ophthalmology, SUNY Downstate Medical Center, Brooklyn, New York, 11203, USA

**^*^** Corresponding author

Yubing Xie, Ph.D., Associate Professor, Colleges of Nanoscale Science and Engineering, SUNY Polytechnic Institute, 257 Fuller Road, Albany, NY 12203

Phone: (518) 956-7381

Fax: (518) 956-8687

Email: [YXie@sunypoly.edu](mailto:YXie@sunypoly.edu)

**Supplementary Information**

Figure Legends

Figure S1. Representative blots of western blot analysis of collagen IV (a and b) in 3D HTM reprobed with house keeping β-actin (c and d). Blots were exposed at several exposure times, e.g., 10 s (a and c), 60 s (b and d).

Figure S2. Representative blots of western blot analysis of fibronectin (a and b) in 3D HTM reprobed with myocilin (c and d), αB-crystallin (e and f), and house keeping β-actin (g and h), respectively. Blots were exposed at several exposu**r**e times, e.g., 10 s (a, c, e and g), 60 s (b, d, f and h).


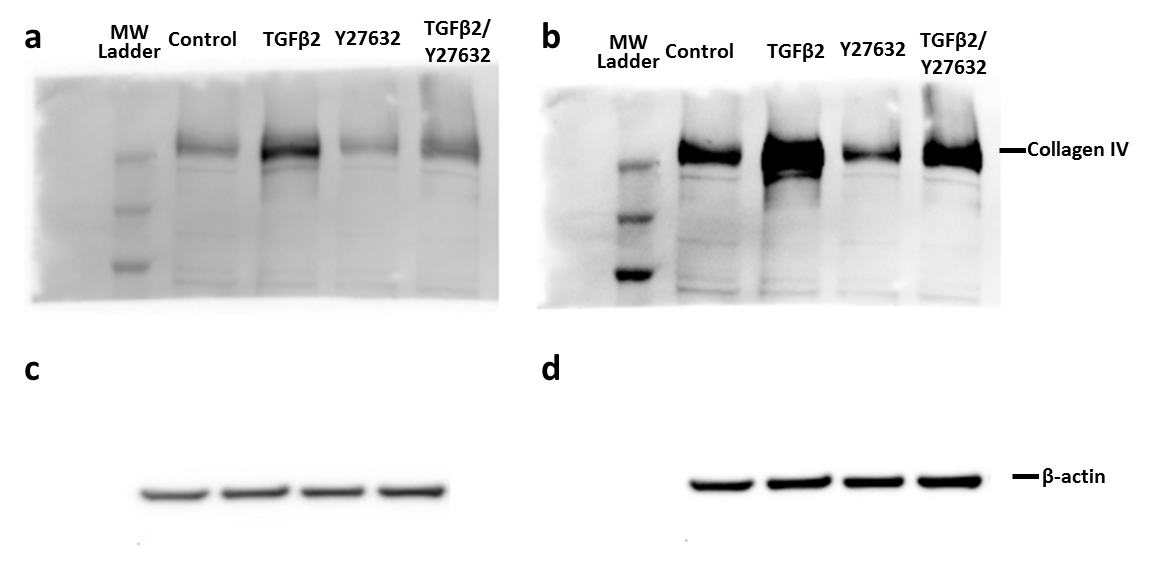


Figure S1


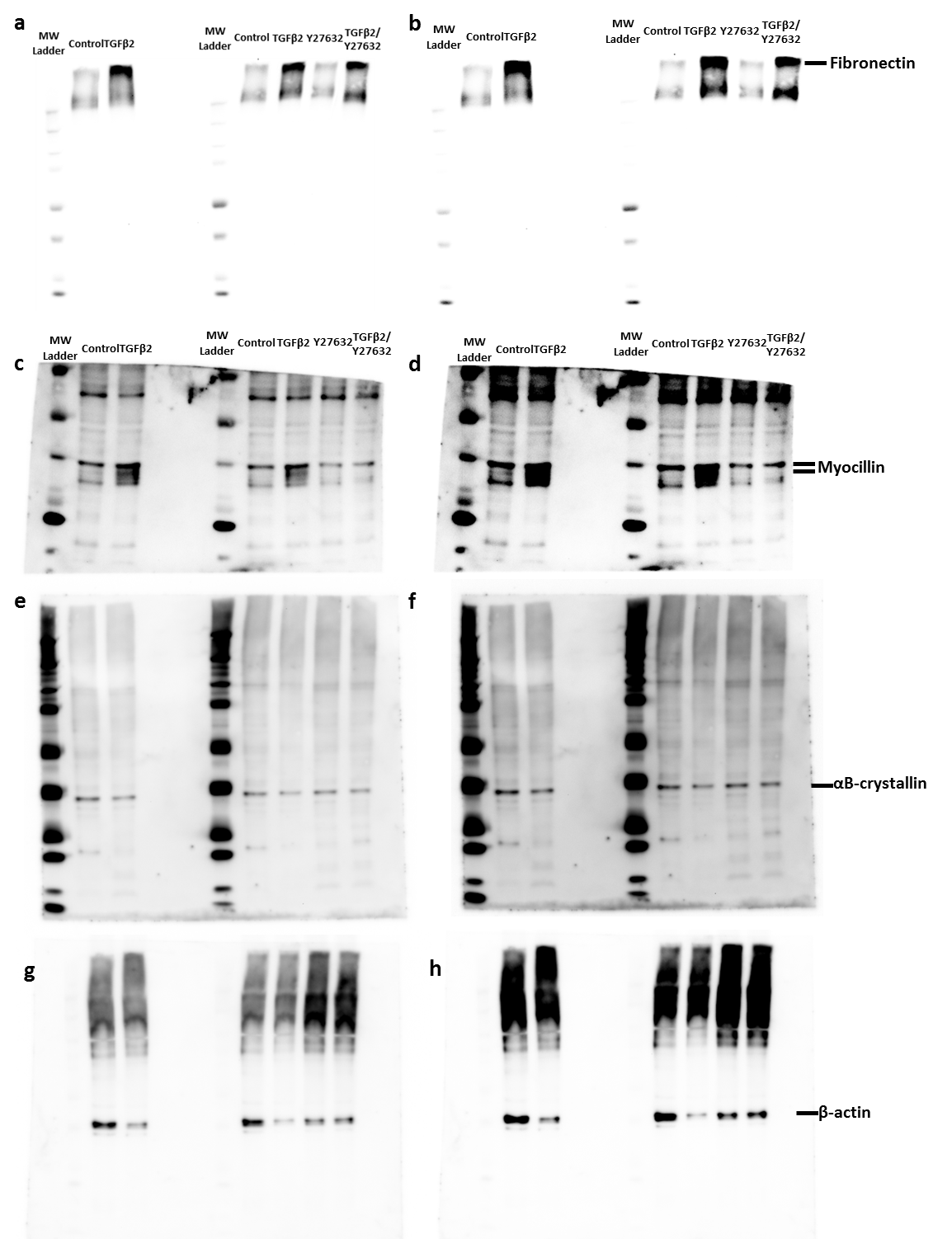
Figure S2
